# Supplementary material for: Theophylline Extracted from Fu Brick Tea Affects the Metabolism of Preadipocytes and Body Fat in Mice as a Pancreatic Lipase Inhibitor
Source: Int J Mol Sci. 2022 Feb 25;23(5):2525. doi: 10.3390/ijms23052525 (PMC8910281; doi:10.3390/ijms23052525)
Supplement: Supplementary file 1 [file ijms-23-02525-s001.zip › ijms-1552782-supplementary.pdf]

International Journal of *Molecular Sciences*

MDPI

Supplement

Theophylline Extracted from Fu Brick Tea Affects the Metabolism of Preadipocytes and Body Fat in Mice as a Pancreatic Lipase Inhibitor

Tian-Tian Liu [1,2], Xiao-Tian Liu [3], Gui-Li Huang [4], Long Liu [1,2], Qing-Xi Chen [3] and Qin Wang [3,*]

1 Science Center for Future Foods, Jiangnan University, Wuxi 214122, China; liutiantian@jiangnan.edu.cn (T.-T.L.); longliu@jiangnan.edu.cn (L.L.)
2 Key Laboratory of Carbohydrate Chemistry and Biotechnology, Ministry of Education, Jiangnan University, Wuxi 214122, China
3 School of Life Sciences, Xiamen University, Xiamen 361005, China; 21620181153704@stu.xmu.edu.cn (X.-T.L.); chenqx@xmu.edu.cn (Q.-X.C.)
4 Agricultural Product Storage and Processing Laboratory, Suzhou Academy of Agricultural Sciences, Suzhou 215105, China; huanggl2015@163.com
* Correspondence: qwang@xmu.edu.cn; Tel./Fax: +86-0592-2185695

Abstract: The dramatic increase in obesity is putting Supplement materils

The crude extract of Fu brick tea was removed by petroleum ether, and then extracted by ethyl acetate and N-butanol. It was found that most of the active substances with inhibitory effect existed in ethyl acetate phase. Figure S1 is the separation and purification flow chart.

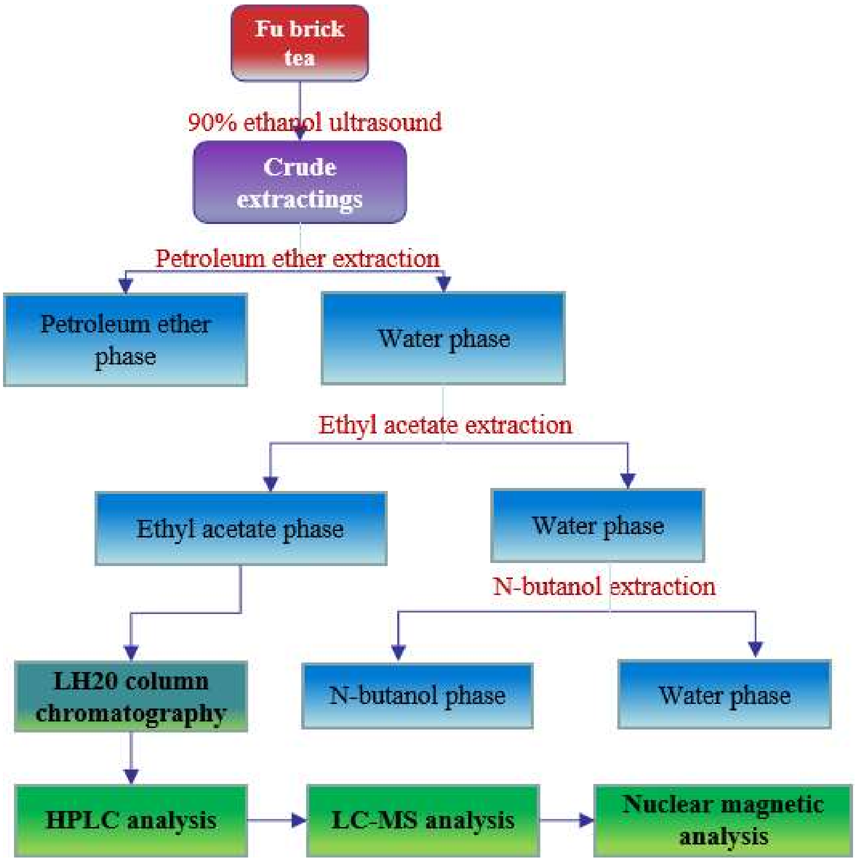

Figure S1. purification process of Fu Brick Tea.

Citation: Liu, T.-T.; Liu, X.-T.; Huang, G.-L.; Liu, L.; Chen, Q.-X.; Wang, Q. Theophylline Extracted from Fu Brick tea Affects the Metabolism of Preadipocytes and Body Fat in Mice as a Pancreatic Lipase Inhibitor. *Int. J. Mol. Sci.* **2022**, *23*, 2525. https://doi.org/10.3390/ijms23052525

Academic Editor: Maurizio Battino

Received: 28 December 2021
Accepted: 6 February 2022
Published: 25 February 2022

Publisher's Note: MDPI stays neutral with regard to jurisdictional claims in published maps and institutional affiliations.

Copyright: © 2022 by the authors. Licensee MDPI, Basel, Switzerland. This article is an open access article distributed under the terms and conditions of the Creative Commons Attribution (CC BY) license (https://creativecommons.org/licenses/by/4.0/).

According to the analysis of previous research results, the crude extract of Fu brick tea with a concentration of 160 mg/mL was preliminarily purified by a hydroxypropyl dextran LH-20 gel column, which is suitable for the separation of hydroxyl-containing substances in organic solvents. Normal phase system ethanol gradient (25%, 50%, 75%, 100%) was used for elution and separation, and small polar hydroxyl-containing molecular substances were collected. The 50% ethanol can enrich most of the target substances. We collected 50% ethanol eluate in batches, and collected it after rotary evaporation and freeze-drying. The yield was 42%.

Next, the enriched Fu brick tea extract was detected by analytical high performance liquid chromatography (HPLC), and compared with the crude extract (Figure S2). the injection concentration was 10 mg/ mL and the injection volume was 200 μL l. Figure S2A was the result chart of Fu brick tea crude extract passing through a HPLC C18 column. the peak shape is very miscellaneous, indicating that there are various substances in it. From the results the figure (Figure S2B) after elution and collection with 50% ethanol on LH-20 gel column, the compound was well separated, with a high main peak, indicating that the main components had been enriched and there were a few miscellaneous peaks, but the peak area was very small and almost negligible, and the elution time was 50 min. as a result, basically no substance was eluted after 35 min. Subsequently, the sample volume was expanded to 1 mL for preparative HPLC collection. After rotary evaporation, freeze drying and storage, the yield was 33.5%.

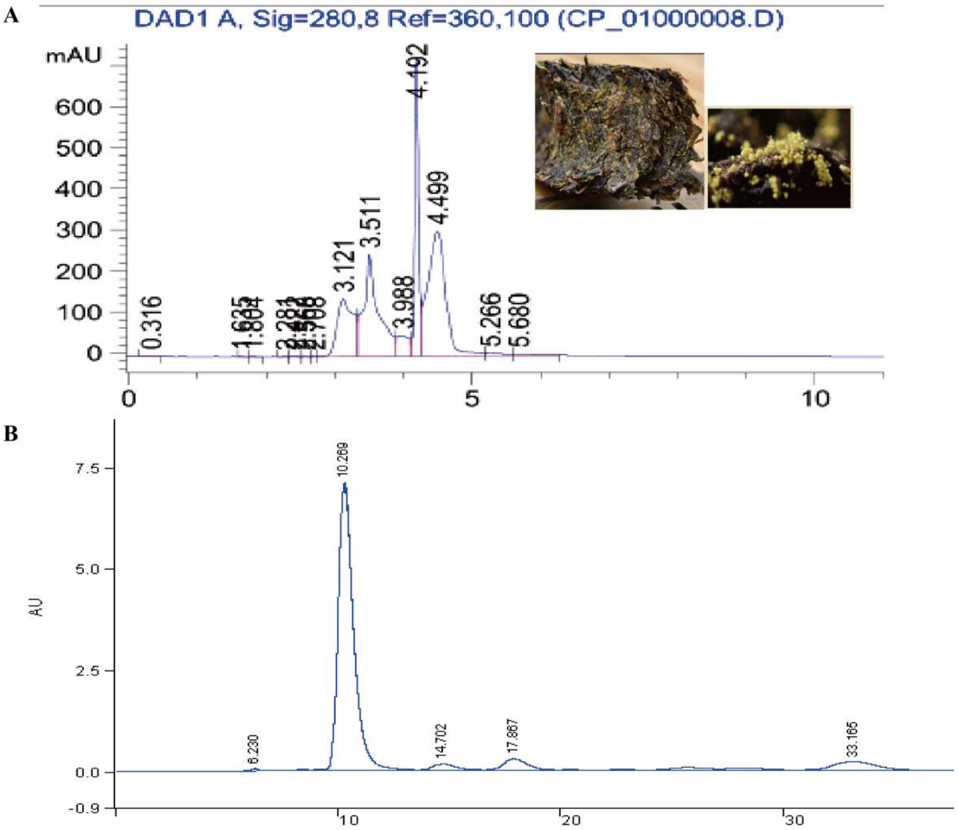

Figure S2. HPLC figure of Fu brick tea. A, HPLC of the ethanol crude extract of Fu brick tea; B, Eluting with 50% ethanol through LH-20 gel column to collect HPLC chromatogram.

With liquid chromatography tandem mass spectrometry (LC-MS/MS) analysis of Fu brick tea extract separated and collected by high performance liquid chromatography (Figure S3) showed that the molecular weight of the enriched substance was mostly 195, got the 98.78% pure with a few other substances or molecular structural fragments. After that, the enriched substance was collected by WATERS 2545 automatic purification

system, and was stored at -80℃ after rotary evaporation and freeze-drying, with a yield of 17.8%.

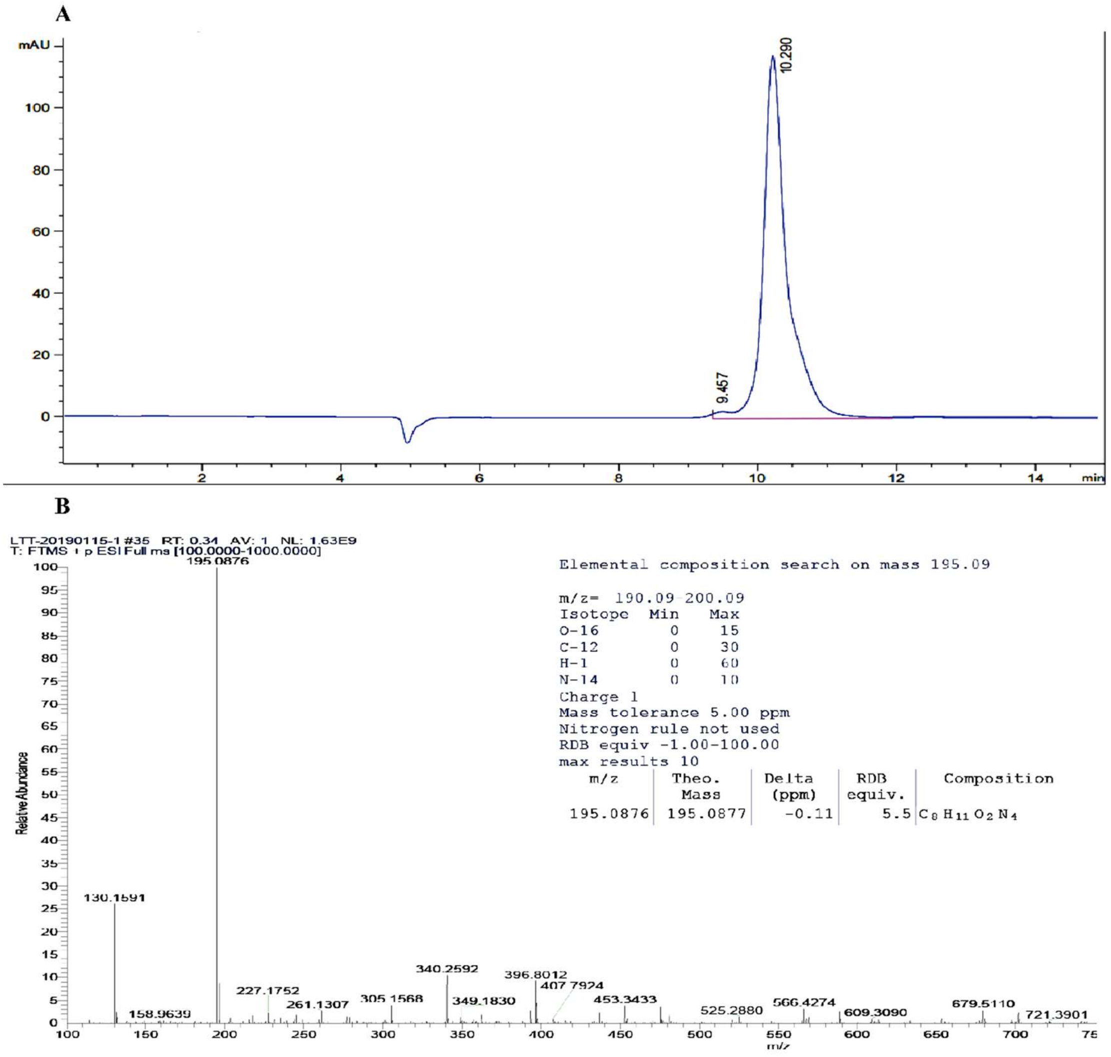

Figure S3. LC - MS/MS spectrometry of Fu brick tea. A, HPLC chromatogram of FBT; B, The mass spectra of FBT.
